# Discriminative Motif Discovery via Simulated Evolution and Random Under-Sampling

**DOI:** 10.1371/journal.pone.0087670

**Published:** 2014-02-13

**Authors:** Tao Song, Hong Gu

**Affiliations:** Faculty of Electronic Information and Electrical Engineering, Dalian University of Technology, Dalian, Liaoning, China; National Institute of Genomic Medicine, Mexico

## Abstract

Conserved motifs in biological sequences are closely related to their structure and functions. Recently, discriminative motif discovery methods have attracted more and more attention. However, little attention has been devoted to the data imbalance problem, which is one of the main reasons affecting the performance of the discriminative models. In this article, a simulated evolution method is applied to solve the multi-class imbalance problem at the stage of data preprocessing, and at the stage of Hidden Markov Models (HMMs) training, a random under-sampling method is introduced for the imbalance between the positive and negative datasets. It is shown that, in the task of discovering targeting motifs of nine subcellular compartments, the motifs found by our method are more conserved than the methods without considering data imbalance problem and recover the most known targeting motifs from Minimotif Miner and InterPro. Meanwhile, we use the found motifs to predict protein subcellular localization and achieve higher prediction precision and recall for the minority classes.

## Introduction

Biological sequence motif is the over-represented pattern in biopolymer (nucleotide or protein) sequences relative to a background model [1]. It is, or is conjectured to be related to the structure and functions of the molecules which the sequences represent. For example, sequence motifs can represent transcription factor binding sites (TFBSs), ribosome binding sites and restriction sites in DNA molecules, splice junctions in RNA molecules, and DNA binding domains (DBDs), post-translational modification sites, protein-protein interaction sites and signal peptide in protein molecules, respectively. So sequence motif discovery algorithm plays an important role in understanding how the cell functions.

Representation of biological sequence motifs can usually be classified into two categories: fixed-length motif representation and variable-length motif representation [1]. The fixed-length motif representation includes: REs (Regular Expressions), PWMs (Position Weight Matrices) and PSSMs (Position-Specific Scoring Matrices), while the variable-length motif representation includes: Profiles, HMMs and profile HMMs [2]. Traditionally, motif finding problem has been dominated by generative models using only the positive class of sequences to produce descriptive motifs of the class, such as MEME [3] using PWMs, and HMMER [2] using profile HMMs. Recently, many studies are focused on discovering of discriminative motifs that occur more frequently in the positive set of sequences and scarcely in the negative set of sequences. When constructing the models, these discriminative methods not only use the information within the positive set of sequences as the traditional generative ones do, but also employ the important information of the negative set. Consequently, the information of the negative set may further refine the models to make the differences between similar classes more obvious.

Up to now, most of the discriminative methods have been used in discovering the DNA motifs. DIPS [4] proposes a probabilistic score to quantify the total number of occurrences of a PWM (motif) in a sequence, defines the objective function to discriminate positive and negative sets of sequences based on this score and uses heuristic hill climbing to search the sequences for motifs that maximizes this function. Seeder [5] is an integrated algorithm that combines the advantages of a pattern-driven method (used in seed PWM searching) and sequence-based method (used in PWM building) and identifies discriminative motifs that are statistically significant (enriched) in the positive set as compared to the background set (negative set). DREME [6] performs a heuristic search of seed motifs represented by REs in the first place, employs a beam search to find motifs represented by PSSMs in the space of seed motifs in the next place and uses the Fisher's Exact Test to compute the significance of the relative enrichment of each motif in both positive and negative set to make the found motifs more statistically significant and discriminative. DECOD [7] represents motifs by PWMs and searches for PWMs that match many k-mers on the positive set while only matching a few on the negative set. Meanwhile, it uses a deconvolution method to accelerate the algorithm, but it does not work well on motifs with large gaps in the middle.

Few investigations have been done on the discriminative motif discovery methods in protein sequences. DEME [8] applies substring retrieval followed by branching discovery to find seed motifs in the global search, refines the data model based on the seed motifs in the local search and combines these to find DNA and protein motifs (represented by PWMs) that optimally discriminate between the positive and negative set of sequences. DiscHMM [9] represents protein sequence motifs by profile HMMs with 'Plan 7' architecture [2] which are considered to be more expressive for protein sequences with insertion and deletion than PWMs, and optimizes the maximal mutual information estimate (MMIE) criterion with extended Baum-Welch algorithm [10], a technique that was primitively applied to speech recognition, to obtain discriminative HMMs. MERCI [11] identifies degenerate motifs based on the candidate generation principle and a given classification of amino acids according to their physico-chemical properties. It introduces two parameters, the minimal frequency threshold for the positive sequences and the maximal frequency threshold for the negative sequences, to find the top K motifs that are most frequent in a positive set of sequences involved in a biological process of interest, and absent from a negative set. DLocalMotif [12] combines three discriminative scoring features: MSC (motif spatial confinement), MOR (motif over-representation), and MRE (motif relative entropy) to determine whether a motif is positioned in a sequence interval in a positive set and is generally absent in a negative set. It first uses a greedy enumeration to generate candidate motifs represented by a triple, and then refines the candidate motifs into a consensus string and subsequently a PWM.

To the author's knowledge, there is little information available in literature about the data imbalance problem in discriminative motif discovery, although it is an important issue in the field of machine learning [13], [14]. The results may be due to (i) most of the benchmark datasets used to discovery motifs, no matter synthetic or real biological, are initially built to be balanced, especially the synthetic ones, although it's actually not the case [9], [15]–[17], (ii) the imbalance problem of sequence data (DNA and protein sequences in motif discovery) are more complex than usual data, consequently, traditional methods for data imbalance problem are no longer applicable. However, some discriminative motif discovery methods, especially those for multi-class classification problem [18], may suffer from the data imbalance problem. For example, DiscHMM tries to find multiple non-redundant targeting motifs for each one of the nine subcellular locations and uses them to convert a new protein into a feature vector for classification, but the precisions and recall rates of relative minority classes, such as secreted, peroxisome and Golgi apparatus, are very low, even zeros. These results indicate that the DiscHMM method is not able to find discriminative motifs for minority classes due to the adverse impact of the data imbalance problem.

In this article, we focus on the data imbalance problem in discriminative motif discovery inspired by DiscHMM which employs MMIE, a discriminative HMM training method, to discover targeting motifs and uses the found motifs for predicting protein subcellular localization. There are two types of data imbalance problems to be solved. The first data imbalance problem is the multi-class imbalance problem that has more than two classes with uneven distributions. The second one relates to the imbalance between the positive and negative datasets at the stage of HMMs training. A lot of methods have been proposed to solve the data imbalance problem such as sampling methods, cost-sensitive methods, kernel-based methods and active learning methods [14]. Among these methods, a sampling method based on over-sampling or under-sampling may be a simple and effective method. Furthermore, it has been empirically evaluated that neither the over-sampling nor the under-sampling alone is always the best one to use, and a combining strategy could be useful and effective [19]. Consequently, we introduce a way to combine the two resampling methods. Firstly, an over-sampling method based on the simulated evolution is applied [15] at the data preprocessing stage. Secondly, a random under-sampling method is employed in the discriminative HMMs training.

A biological benchmark dataset has been used to characterize the efficiency of our method compared with other methods. The results indicate that our method has superior precision and recall for minority classes because of its ability to use phylogenetic knowledge imported by simulated evolution and information in the negative training set. In addition, our method recovers the most known targeting motifs from Minimotif Miner [20] and InterPro [21] and discovers more novel conserved motifs than the methods without considering the data imbalance problem. Furthermore, we believe that the strategy of solving the data imbalance problem proposed in this article is useful for further studies on discriminative motif discovery of multi-class protein sequences.

## Methods

Our method is described below in the following aspects: problem formulation, combining strategy of sampling, protein subcellular localization prediction, datasets and implementation.

### Problem Formulation

We represent the motifs by HMMs and use the following notations: 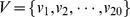 denotes the 20 kinds of amino acids. The training sequences are given as 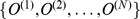, where 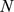 is the number of training sequences. 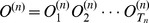, where 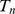 is the length of 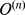 and 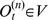, 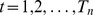, 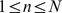. Let the class labels of the sequences be 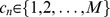, 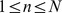. The HMM for the 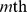 class is denoted as 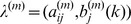, where 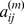 and 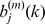 represent the transition and emission probability of 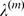 respectively.

Generative training of HMMs is to optimize the maximum likelihood estimation (MLE) criterion with the Baum-Welch algorithm [22]. The MLE objective function is given by the following formula:
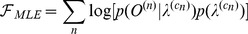
(1)


While discriminative training of HMMs is to optimize the MMIE criterion with the extended Baum-Welch algorithm [10]. The MMIE objective function can be defined as follows:
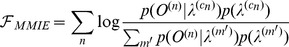
(2)


The assumption of one occurrence per sequence (OOPS) model [23]–[25] for motif occurrences per sequence is used and motifs are found on flat and hierarchical structure of compartments (see Figure S1 in File S1) similar to the approach taken by DiscHMM, where the hierarchical structure is a tree structure that mimics cellular sorting pathways [26] and it can provide some prior knowledge for motif discovery and prediction of protein subcellular localization which the flat structure cannot. When training HMMs by MMIE, the positive and negative training sets need to be used at the same time, so the one-vs.-all strategy [27] is adopted. For the flat structure, the negative training set is picked as the union of all the class sets except the positive one. While for the hierarchical structure, the negative training set is selected as the union of all the class sets except the positive one under the root node or the same intermediate node. Additional details of motif finding on flat and hierarchical structure of compartments are provided in [9].

After a motif (represented by HMM) is found, top motif instances will be retrieved from protein sequences by posterior decoding: for each candidate motif, we scan all protein sequences, identify all possible positions (motif instances) of the begin state and the end state of the motif and compute the product of the posterior probabilities of the begin state and the nearest end state for ranking. Then, all positions in all sequences are ranked by this product. Finally, when the number of the top positions is given, they are selected according to the ranking.

### Combining Over-sampling with Under-sampling to Deal with Data Imbalance

The targeting motifs discovered by DiscHMM are more discriminative according to their effect in the overall accuracy of predicting protein subcellular localization than the generative motifs. However, the precision and recall of the relative minority classes are very low, even zeros. Therefore, a combining strategy of resample is adopted to solve the data imbalance problem. This strategy applies the over-sampling method via simulated mutation to generate new sequences for the minority classes and random under-sampling method in MMIE for HMM training.

### Over-sampling via Simulated Evolution

The first kind of data imbalance problem is the multi-class imbalance, which is the main reason for the low precision and recall of minority classes. To solve this problem, we try to use over-sampling method to add new sequences into the minority classes. In order to avoid over-fitting, we do not use the random over-sampling method that randomly selects sequences from the minority class sets, replicates them and adds the replicated ones into the original sets [28]. Besides the random over-sampling method, there are no suitable over-sampling methods for protein sequences, since most of the over-sampling methods in the field of machine learning such as SMOTE [29] are not designed for sequence data. So in this paper, we intend to employ simulated evolution [15]-[17], an over-sampling method that incorporates phylogenetic knowledge and has been successfully applied to classify protein families and recognize beta-structural motifs.

We use the BLOSUM (BLOcks SUbstitution Matrix) matrix as the model of evolutionary mutations. The BLOSUM matrix is a substitution matrix used to measure the similarity between protein sequences for alignment of them. BLOSUM matrices are derived from about 2000 blocks of aligned sequence segments characterizing more than 500 groups of related proteins [30]. We conduct an experiment to test BLOSUM45, BLOSUM62 [31] and BLOSUM80 for simulated mutation. The results (see Figure S2 in File S1, Figure S3 in File S1 and Figure S4 in File S1) empirically demonstrate that substituting amino acid residues in the simulated mutation through BLOSUM62 may ensure both the conservation and diversity of the generated sequences, so that we can not only make the found motifs evolutionary conserved, but also get multiple non-redundant motifs that are useful for predicting protein locations. Detailed discussions are provided in Supporting Information. Consequently, we choose the BLOSUM62 matrix as the model of simulated mutations. Our purpose is to make the severely skewed class distribution to be uniform. To achieve this goal, the simulated evolutionary approach is used to make the number of training sequences in every class the same as the one of the largest class set. The pseudo-code for simulated evolution is shown as follows.

### Simulated Evolution algorithm

**Input:** Training sequences 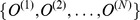


Sizes of classes 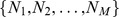


Mutation rate 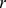, 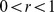


Calculate the largest size of classes 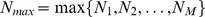



**For** 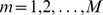


**For** 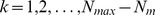, **do**

1. Randomly select a sequence 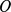 from class 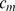

2. Obtain the length 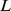 of 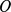 and the number 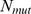 of amino acids need to be mutated in 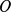, where 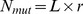

3. Randomly pick 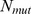 positions in 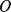

4. Replace the amino acid in every one of the 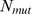 positions with another one that has the highest probability value in the BLOSUM62 matrix
5. Add the new sequence into the set of class

**Output:** The new training datasets

### Random Under-sampling for MMIE

After building the new training datasets by simulated evolution, we can use it and the extended Baum-Welch algorithm to optimize the MMIE criterion to get discriminative motifs represented by HMMs. Although the found motifs by the above methods are discriminative and useful for classification, both the percentage and significance of the conserved motif instances retrieved by those HMMs are low, which will be described in details later. The results may be attributed to the one-vs.-all strategy adopted for MMIE, since we have used the simulated evolution method to generate so many new sequences and the negative training set becomes so large that the extended Baum-Welch algorithm is affected.

Therefore, the random under-sampling method is applied to make the size of negative training set as large as the positive one. Moreover, we don't intend to randomly select a set of the original negative set before the training of HMMs as usual machine learning methods do, which may cause the extended Baum-Welch algorithm to miss important sequences of the negative set resulting in less discriminative HMMs [14]. In order to fully exploit the useful information in the negative training set and prevent the loss of important negative samples, a set of the original negative set is randomly selected at each iteration of the extended Baum-Welch algorithm. The number of the negative sequences selected at each iteration is the same as that of the positive ones. However, this simple trick has achieved very good results which will be shown and discussed later.

### Protein Localization Prediction and Motif Ranking

The main evaluation of the discovered motifs is based on their classification performance, so the log-likelihood ratio [32] is used as the feature and the support vector machine classifier (SVM) as the base classifier for classification. The log-likelihood ratio is shown as the formula (3), where 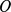 is a new sequence needed to be classified, 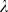 represents one of the multiple non-redundant motifs (HMMs) of a certain class and 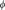 denotes the background model or known as null model which is selected as the default one in HMMER. The SVM is used with the flat and hierarchical structure and a 10-fold cross-validation procedure is carried out that the proposed method are trained on part of the sequence set and tested on proteins not used to learn the motifs.
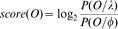
(3)


The found motifs are ranked by using one-step backward feature selection to further evaluate their contributions for predicting subcellular localization. For the SVM and the feature vector consisting of log-likelihood ratios, the accuracy after removing each feature (corresponding to a motif) is recorded and the motifs are ranked according to the ascending sort of the accuracies.

### Datasets

We apply our discriminative motif discovery method via simulated evolution and random under-sampling to the PSLT2 dataset [33]. This dataset consists of 1,521 *Saccharomyces cerevisiae* proteins with curated localization annotation in UniProt [34] and several proteins are localized to more than one compartment. Localization annotations described as “possible,” “potential,” “probable,” or “by similarity” are excluded, so the rest of the localization annotations are kept that clearly indicated which compartment(s) the protein is localized to. Excluding the proteins with multiple localizations, the number of proteins with single localization is 1,208 denoted by dataset I. Assigning the proteins with multiple localizations to their corresponding compartments, the total number of sequences is 1,889 denoted by dataset II. Proteins are annotated with nine labels: nucleus, cytosol, peroxisome, mitochondria, endoplasmic reticulum (ER), Golgi apparatus, vacuole, plasma membrane, and secreted. The sizes of these nine classes in the dataset I and dataset II are presented in Table 1.

**Table 1:** Compartment distribution.

Compartment	Num.I	Percent.I	Num.II	Percent.II
Cytosol	190	15.73	453	23.98
ER	84	6.95	156	8.26
Golgi	25	2.07	77	4.08
Vacuole	30	2.48	43	2.28
Mitochondria	312	25.83	345	18.26
Nuclear	454	37.58	647	34.25
Peroxisome	17	1.41	24	1.27
Membrane	86	7.12	126	6.67
Secreted	10	0.83	18	0.95
Total	1208	100	1889	100

This table shows that the compartment distribution is severely skewed. The first column is the cellular compartment; the second and third columns are the number and percentage of sequences in each cellular compartment of the dataset I, while the fourth and fifth columns are those of the dataset II.

### Implementation

Five sets of experiments are implemented: (i) generative training of HMMs, (ii) discriminative training of HMMs, (iii) generative training of HMMs with simulated evolutionary mutation, (iv) discriminative training of HMMs with simulated evolutionary mutation, (v) discriminative training of HMMs with simulated evolutionary mutation and under-sampling. In addition, all the algorithms above are executed on the flat and hierarchical structure respectively. For the sake of clarity, the above algorithms are marked as Gen, Disc, GenM, DiscM and DiscMU respectively. The evolutionary mutation rate for GenM, DiscM and DiscMU is selected as 20% as it has been shown in [15] that a mutation rate around 20% performs better for building profile HMMs to recognize G-protein coupled receptor proteins in different classes and SCOP super-family proteins from different families.

To make a fair comparison, we set similar options for all five algorithms: motif length is set to 4 that the number of match states is 4 for HMM. For generative HMM (Gen and GenM), we run the Baum-Welch algorithm with 10 random initialization and at most 50 iterations. For discriminative HMM (Disc, DiscM and DiscMU), the generative HMM algorithm is executed firstly with the same options described above, then the extended Baum-Welch algorithm is implemented on the HMM with highest likelihood with 50 iterations at most. In order to obtain multiple non-redundant motifs for each compartment, after a motif is found we mask the amino acids assigned to match states by Viterbi algorithm [35], [36] with random amino acids, and the process is repeated 10 times.

The reasons for setting the motif length to be 4 are: firstly, the average length of functional eukaryotic linear motifs validated experimentally, including ligand sites, post-translational modification sites, proteolytic cleavage and processing sites and subcellular targeting sites, is approximately 6 (6.3) residues. Simultaneously, the 6.3 residues contain 3.7 defined positions (positions that cannot tolerate an amino acid substitution or can tolerate a limited number of amino acid substitutions that usually share some physicochemical or structural property) on average [37], and the concept of defined position is consistent with the match state of HMM. Secondly, it has been experimentally validated that when comparing setting the length of HMM to 4 versus setting the length to range from 3 to 7, the performance of predicting protein locations doesn't fluctuate much [38].

The SVM is trained and tested by the software SVMlight [39] with the linear kernel. The default options are used and the value for the trade-off between error and margin is set to 0.01 as in the DiscHMM. The simulated evolution is implemented in MATLAB and the new datasets are stored before HMM training. Execution of the discriminative HMMs training with random under-sampling method is based on the DiscHMM source code.

## Results

We compare the results of the five sets of experiments with the same evaluation criteria as the DiscHMM and focus on the impact of the data imbalance on the motif discovery and classification. In order to prevent the impact brought by excessively high sequence similarity, all the results are obtained on the original PSLT2 dataset not the augmented dataset after simulated mutation.

### Combining Resampling Improves Prediction Precision and Recall for Minority Classes

The primary goal of our work is to identify novel discriminative targeting motifs which are occurring frequently in the positive set while scarcely or not occurring in the negative set. Since there is still a little current knowledge about targeting signals, finding a discriminative targeting motif based on its effect of classifying positive and negative sequences may be a useful and effective way. As a result, we use the found motifs to predict localization and further apply the prediction results to evaluate motif finding methods. The evaluation criteria used here are recall, precision and accuracy, and they are given by the following formulas:
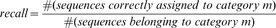
(4)




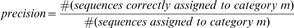
(5)




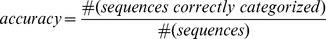
(6)


**Figure 1:**
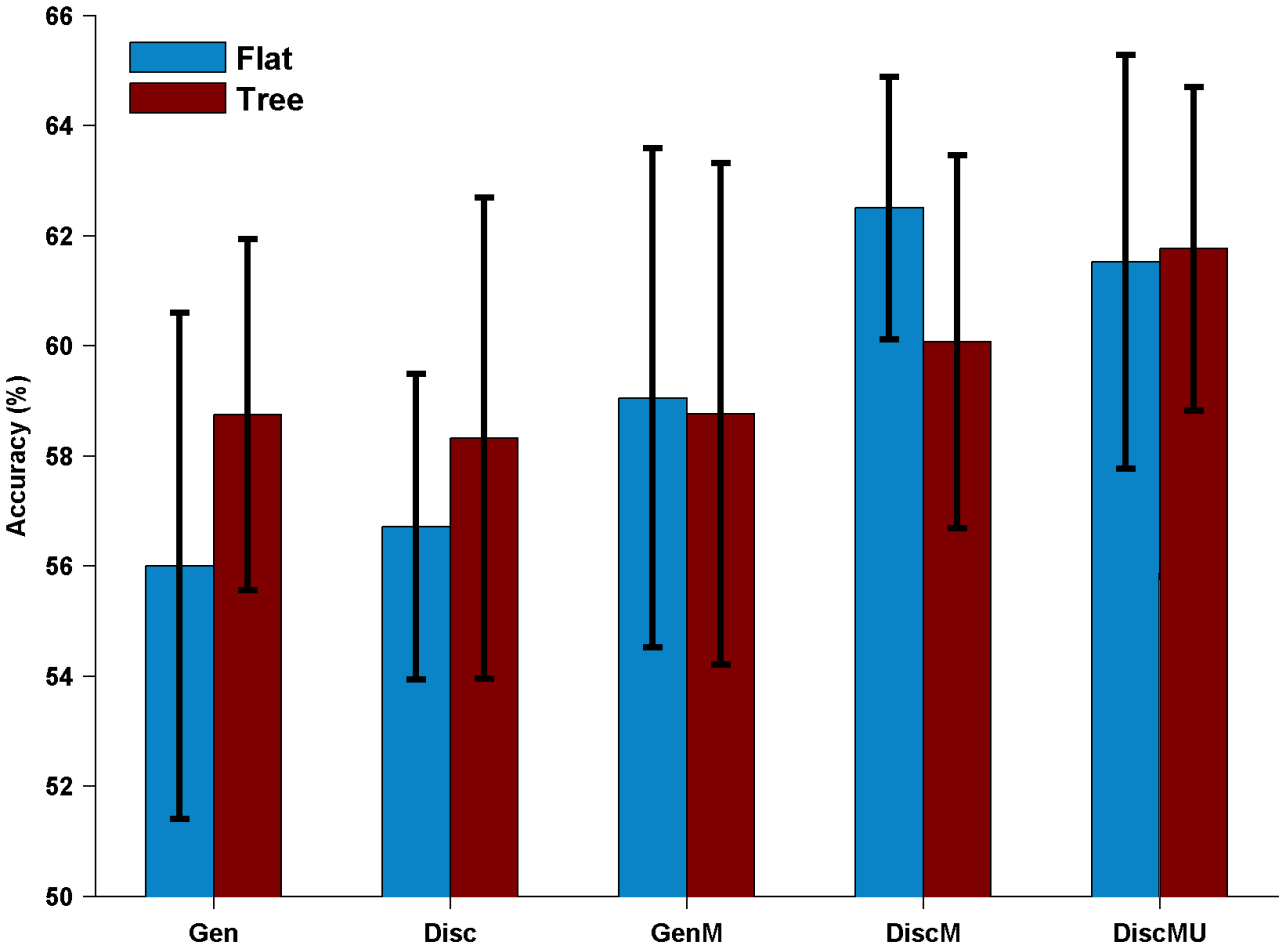
Total accuracy of predictions. Motifs are discovered by the five methods on flat and hierarchical (tree) structure respectively.

The overall accuracies of this prediction comparison are shown in Figure 1. From this figure we can get following conclusions:

1. The total accuracy of GenM, DiscM and DiscMU are higher than Gen and Disc, this is probably due to the application of over-sampling via simulated evolution, as a result, the multi-class imbalance problem has been solved, so that the precision and recall of minority classes increase substantially which will be described in detail later.
2. The hierarchical structure, that mimics the cellular sorting pathways and may provide another layer of biological information, leads to great improvement in classification results for Gen and Disc which has been shown in DiscHMM, but it has a little effect on DiscMU and even worse than flat structure for GenM and DiscM. The reasons for the above situations may be that we use over-sampling method based on a simple simulated evolutionary model to generate new sequences and don't consider embodying the information of the cellular sorting pathways in that model, therefore, some of the new sequences may contain more targeting information of the negative class than their own class, so that it is affected when using the hierarchical structure of compartments for training and classification. These have greater impact on GenM and DiscM than DiscMU since in DiscMU we use under-sample to reduce this impact to some extent.
3. The method Disc doesn't outperform Gen as it is presented in [9], although only a few differences between our experiments and DiscHMM in the experimental parameter settings such as the number of random initializations reduces from 100 to 10 and the maximum iterations for both generative and discriminative training reduce from 100 to 50. The reason may be that no matter the Baum-Welch algorithm for MLE or the extended Baum-Welch algorithm for MMIE, both of them are based on the EM (expectation-maximization) algorithm which is easy to fall into local optimum when choosing an inappropriate initial estimates of the HMM parameters [22]. So this result may be caused by the reduction of initializations.
4. GenM and DiscM, versions of Gen and Disc respectively combining over-sampling, receive high prediction accuracies. Specifically the DiscM with flat structure which reaches the highest accuracy (62.5%). However, both the percentage and significance of the conserved motif instances discovered by these two methods are lower than Gen and Disc which will be shown in detail later. This indicates that the motifs found by these two methods are very helpful in classifying, but them may be not potential sorting signals or involved in other functions that proteins in a given compartment need to carry out. Further, we will discuss later that the motifs found by DiscMU method, versions of Disc combining over-sampling and under-sampling, are not only beneficial to classification, but also can find more conserved motifs.

The above discussions on overall accuracy don't adequately reflect the negative impact of data imbalance problem and the superiority of our algorithm. Therefore, we do a further test on dataset I to illustrate the performance of Disc and DiscMU on each compartment. The results are displayed in Table 2 and Table 3, where parenthesis after the rows are percentage of labels.

**Table 2:** Precision of each compartment.

Compartment	Disc	Disc	DiscMU	DiscMU
	flat (%)	tree (%)	flat (%)	tree (%)
Secreted (0.8)	3.33	0.00	36.00	41.67
Peroxisome (1.4)	0.00	0.00	66.67	50.00
Golgi (2.1)	28.33	10.00	51.50	59.17
Vacuole (2.5)	15.00	25.00	40.00	57.50
ER (7.0)	49.98	62.35	55.52	64.94
Membrane (7.1)	67.13	59.30	58.69	66.26
Cytosol (15.7)	54.56	50.26	71.99	59.44
Mitochondria (25.8)	50.66	48.93	56.52	51.28
Nuclear (37.6)	63.24	70.42	65.96	71.39

This table shows that our method significantly improves the precision of the minority classes.

**Table 3:** Recall of each compartment.

Compartment	Disc	Disc	DiscMU	DiscMU
	flat (%)	tree (%)	flat (%)	tree (%)
Secreted (0.8)	10.00	0.00	34.33	35.67
Peroxisome (1.4)	0.00	0.00	55.00	34.17
Golgi (2.1)	4.68	1.00	20.55	13.47
Vacuole (2.5)	5.33	8.67	25.69	32.43
ER (7.0)	29.90	28.27	36.42	35.38
Membrane (7.1)	35.14	43.30	27.05	34.32
Cytosol (15.7)	14.97	29.45	18.16	32.88
Mitochondria (25.8)	64.04	67.41	70.61	72.99
Nuclear (37.6)	73.62	64.78	73.36	62.43

This table shows that our method significantly improves the recall of the minority classes.

As we can see, the precision and recall of almost all compartments are improved by our method, except the precision of Membrane (7.1) reduces from 67.13% by Disc on flat structure to 66.26% by DiscMU on tree structure, the recall of Membrane (7.1) reduces from 43.30% by Disc on tree structure to 34.32% by DiscMU on tree structure and the recall of Nuclear (37.6) reduces from 73.62% by Disc on flat structure to 73.36% by DiscMU on flat structure. However, the precision and recall of the relatively minority classes, such as Secreted (0.8), Peroxisome (1.4) and Golgi (2.1), improve greatly. The results imply that the discriminative HMMs training with the strategy of combining over-sampling with under-sampling is an effective method for protein subcellular localization prediction.

**Figure 2:**
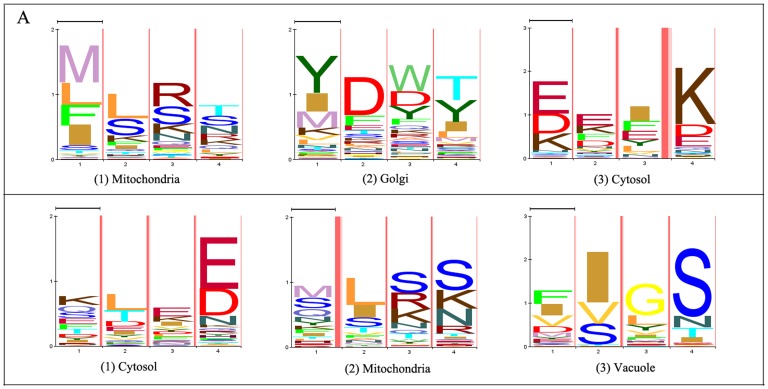
Top 3 motif candidates. These motifs are most predictive of localization, which are discovered on hierarchical compartment structure by (A) Disc and (B) DiscMU. The x-axis title of each HMM logo is the rank and compartment of the motif.

The three most discriminative motifs found by Disc and DiscMU using hierarchical compartment structure are shown in Figure 2. The discriminative motifs are ranked by one-step backward feature selection as described before. Motifs are visualized using HMM logos [40]. A stack of letters represents a match state, the total stack height is the relative entropy between the match state's emission distribution and the background distribution of letters, the relative height of each letter in the stack is proportional to its emission probability. A red-shaded stack visualizes an inserted state, where widths of dark and light part correspond to the hitting probability and the expected length respectively.

### Over-sampling via Simulated Evolution Helps to Recover Known Targeting Motifs

After evaluating the found motif by precision and recall of localization prediction, we would like to identify how many of motifs discovered by different methods were previously known.

A list of known targeting motifs collected by Lin [9] is used in this paper. This list includes 56 known targeting motifs, 23 of them from Minimotif Miner and 33 from InterPro, where the InterPro motif may be associated with two or three distinct localizations (see Table S2 in File S1). A comparison of found motif instances with known motif instances is executed to determine whether a known targeting motif is recovered. For instances of a known motif, retrieving top instances of the found motifs by posterior decoding as described above where the number of found motifs' instances is four times the number of the known motif's instances, the evaluation criterion of whether the known motif is recovered is that one-third of its instances are overlapped by the instances of the found motifs at least half the motif length.

**Figure 3:**
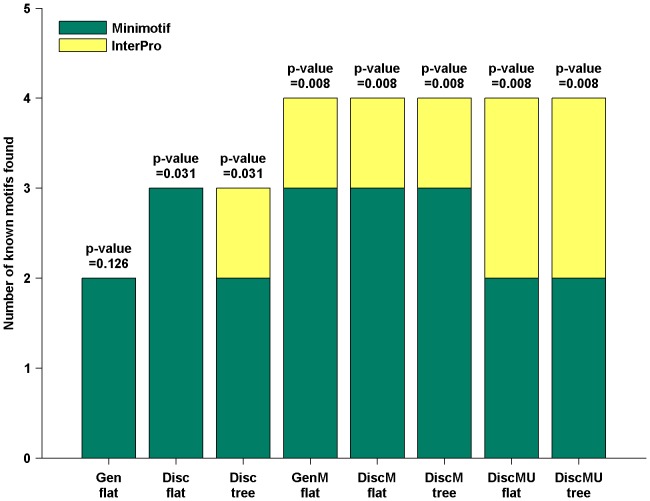
Number of known motifs recovered by different methods. The p-values are calculated by generating random motifs.

Figure 3 shows the number of known motifs recovered by different methods and their corresponding p-value used to estimate statistical significance which is calculated by generating random motifs (for how to calculate the p-value, please see the Supporting Information for details). As we can see, those motif discovery methods in which we employ simulated evolution (GenM, DiscM and DiscMU) were able to identify the most motifs followed by Disc. The reasons to explain this result are the sample diversity and phylogenetic knowledge brought by simulated mutation. The simulated evolution generates a lot of new training sequences integrated with phylogenetic knowledge by using the information in the BLOSUM62 matrix, this may make our method conducive to identify the known targeting motifs. Moreover, DiscMU, in which we incorporate simulated evolution and under-sampling into the discriminative HMM training method, finds the same number of motifs in known targeting motif database Minimotif Miner and InterPro while other methods recover few motifs in InterPro than Minimotif Miner. This may imply that DiscMU is not susceptible to the influence of the difference between databases when recovering known targeting motifs. A comparison of the HMM logos of the motifs found by DiscMU and the regular expressions of known motifs recovered is provided in Table S1 in File S1 and Table S3 in File S1.

### Combining Resampling Improves Percentage and Significance of Conserved Motifs

The previous discussion shows that methods with over-sampling by simulated evolution were able to recover the most known motifs. However, they recover only a few known targeting motifs, even the most effective method recovers only 4 from 56. This is probably due to that there have been many real targeting motifs that are unknown up to now. Moreover, since the found motifs are very useful for protein subcellular localization predicting, some of them may play an as yet unidentified role in localization or other different biological interpretations of sequence signatures that differentiate between proteins that reside in different compartments. The key problem is how to validate them as potential sorting signals or involved in other functions that proteins in a given compartment need to carry out. One way is using the analysis of motif conservation: the found motifs that are expected to be potential sorting signals are more conserved among evolutionarily close species [41].

First, according to *Saccharomyces* Genome Database (SGD) fungal alignments [42] for seven yeast species [43], [44], each amino acid in the 1,521 *Saccharomyces cerevisae* protein sequences is defined as four conservation states: no conservation versus weak, strong, and identical conservation (across seven species). Second, for each of the five methods, 20 most discriminative motif candidates defined by backward feature selection as described previously are chosen. Third, for each of the 20 motifs, the top 30 positions based on likelihood or posterior probability are retrieved. Finally, for each motif instance, it is considered conserved if all sites in that instance are labeled as having strong or identical conservation by ClustalW [45].

**Figure 4:**
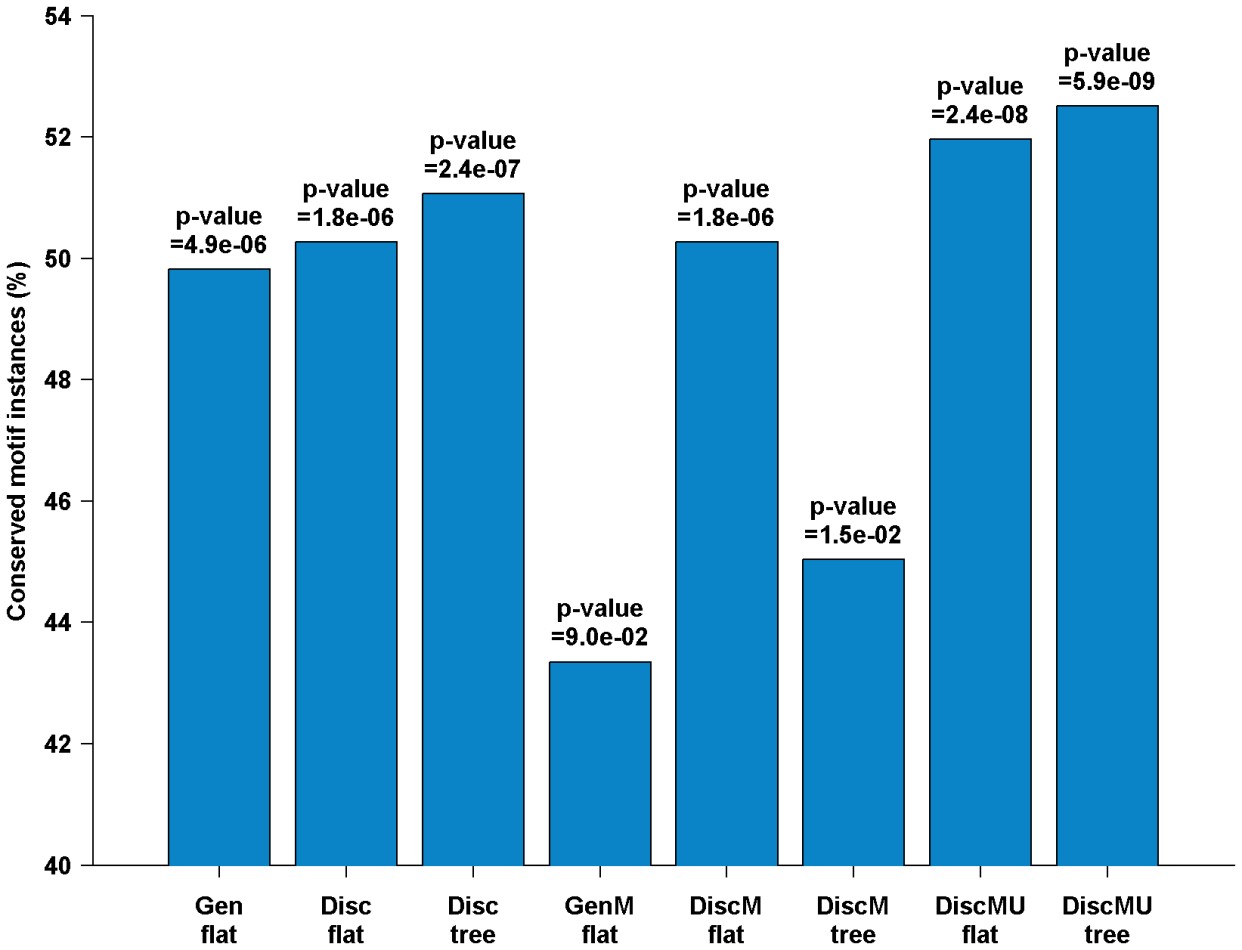
Percentage of conserved instances of the top 20 candidate motifs. The p-values are calculated by hypergeometric test.

The percentage of conserved motif instances for different methods and the corresponding statistical significance are presented in Figure 4 (for how to calculate the p-value, please see the Supporting Information for details). The percentage of conserved 4-mers is only 41 [9]. In contrast, for motifs identified by DiscMU using flat or hierarchical structure, 52 percent and 53 percent of motif instances are conserved, respectively. For Disc using flat or hierarchical structure, 50 percent and 51 percent of motif instances are conserved, respectively, and for Gen, 50 percent instances are conserved. For DiscM using flat or hierarchical structure, 50 percent and 45 percent of motif instances are conserved, respectively, and for GenM, 43 percent instances are conserved. The conservation achieved by DiscMU using hierarchical structure is the highest among the methods.

These results clearly indicate that motif instances discovered by all methods are significantly conserved when compared to conserved 4-mers. Moreover, For GenM and DiscM, versions of Gen and Disc respectively combining over-sampling, both the percentage and significance of the conserved motif instances are lower than Gen and Disc, although they are very useful in predicting locations. As we mentioned before, since we have used the simulated evolution method to generate a large number of new sequences, when we adopt the one-vs.-all strategy for MMIE, the negative training set becomes so large that the extended Baum-Welch algorithm is affected for training HMM. Consequently, HMM trained by GenM and DiscM cannot reflect the common characteristics of the positive training sequences. However, DiscMU, incorporating simulated mutation and random under-sampling into discriminative HMM training, achieves relatively high percentage, significance of the conserved motif instances and prediction accuracy. This suggests that the proposed method is not only contributing to classify proteins, but also more useful for discovering conserved motifs.

## Discussion

We have developed and used a new method that applies a simple strategy of combining over-sampling with under-sampling method in discriminative HMM training to discover protein targeting motifs. The combining strategy contains over-sampling method via the simulated evolution for multi-class imbalance problem at the data preprocessing stage and random under-sampling method for the imbalance between the positive and negative datasets at discriminative HMM training stage. It successfully deals with these data imbalanced problems and assists in identifying more discriminative motifs in terms of prediction precision and recall for all compartments especially the minority classes. According to the conservation analysis, most of the novel motifs discovered are considered to be potential sorting signals or involved in other functions that proteins in a given compartment need to carry out. The advantage of our method is probably due to two aspects: first, the over-sampling method via simulated evolution contains phylogenetic knowledge obtained from the information in the BLOSUM62 matrix [15], while avoiding over-fitting as a result of the diversity of new generated sequences. Second, while guaranteeing the full use of useful information in negative training set, the random under-sampling method reduces the influence of the excessive negative training sequences.

Our method provides a way to solve the data imbalance problem in discriminative motif discovery with multiple-class. Although we take the discovery of protein targeting motifs as an example to show the power of our method, our method can be easily applied to other problems of motif discovery and can contribute significantly to finding efficient, discriminative and conserved motifs, e.g., DBDs, post-translational modification sites and protein-protein interaction sites.

Although the results presented in our paper show that the proposed method is relatively efficient in solving the data imbalance problem in discriminative motif finding, there are still same inadequacies in our work. For example, we select the 20% mutations in our experiment since it performs better for building profile HMMs to recognize G-protein coupled receptor proteins and SCOP super-family proteins, however, it is very important to research on how different mutation rates affect the establishment of profile HMMs for targeting motifs and how to build different mutation models for different subcellular compartments, such as the *β*-Strand mutation model for beta-structural motifs [16], to avoid destroying the invariant amino acids of targeting motif instances. In addition, the motif length is set to 4 for HMM training in our experment, but when we compare the known motifs and found motifs, it indicates that when the length of the known motifs is around 4, the regular expressions of the known motifs recovered match the found HMMs relatively better, otherwise they are not good and especially when the length of the known motif is particularly long (see Table S1 in File S1 and Table S3 in File S1). Therefore, finding the optimal motif length is very important in motif discovery and we will consider this problem in future research.

In addition to the cases mentioned above, it is helpful to take advantage of the attributes of short linear motifs [37] to limit the motif discovery intervals in protein sequences and reduce the computational complexity of the Baum-Welch algorithm [46], so that the proposed approach can be applied on large datasets more effectively. Meanwhile, for future work, we would like to incorporate some known information of subcellular localization into the HMM as the prior knowledge to make the targeting motifs more precise and introduce self-adaptive sampling method according to the weights of sequences instead of treating all sequences equally to make the found motifs more discriminative in HMM training.

## Supporting Information

File S1 **Supporting Information. Figure S1.** Hierarchical (tree) structure of compartments based on cellular sorting. **Figure S2.** Accuracy of predictions based on simulated evolution by using BLOSUM45, BLOSUM62 and BLOSUM80 respectively. **Figure S3.** Number of known motifs recovered based on simulated evolution by using BLOSUM45, BLOSUM62 and BLOSUM80 respectively. **Figure S4.** Percentage of conserved instances of the top 20 candidate motifs based on simulated evolution by using BLOSUM45, BLOSUM62 and BLOSUM80 respectively. **Table S1.** The known motifs of InterPro recovered by DiscMU. **Table S2.** The sequences that contain IPR00426. **Table S3.** The known motifs of Minimotif Miner recovered by DiscMU.
(DOC)
