# Supplementary material for: Discriminative Motif Discovery via Simulated Evolution and Random Under-Sampling
Source: PLoS One. 2014 Feb 13;9(2):e87670. doi: 10.1371/journal.pone.0087670 (PMC3923751; doi:10.1371/journal.pone.0087670)
Supplement: File S1 — Supporting Information. Figure S1. Hierarchical (tree) structure of compartments based on cellular sorting. Figure S2. Accuracy of predictions based on simulated evolution by using BLOSUM45, BLOSUM62 and BLOSUM80 respectively. Figure S3. Number of known motifs recovered based on simulated evolution by using BLOSUM45, BLOSUM62 and BLOSUM80 respectively. Figure S4. Percentage of conserved instances of the top 20 candidate motifs based on simulated evolution by using BLOSUM45, BLOSUM62 and BLOSUM80 respectively. Table S1. The known motifs of InterPro recovered by DiscMU. Table S2. The sequences that contain IPR00426. Table S3. The known motifs of Minimotif Miner recovered by DiscMU. (DOC) [file pone.0087670.s001.doc]

Supporting Information for

Discriminative Motif Discovery via Simulated Evolution and Random Under-sampling

Tao Song^1^, Hong Gu^1, ∗^

1 Faculty of Electronic Information and Electrical Engineering, Dalian University of Technology, Dalian, Liaoning, China

∗ E-mail: guhong@dlut.edu.cn

1 Performance under Different Scoring Matrices

Experiments are conducted to test BLOSUM45, BLOSUM62 and BLOSUM80 for simulated mutation under 20% mutations. The evaluation criteria are classification accuracy, known motifs recovery and conservative analysis. All the results are obtained on the original PSLT2 dataset by using DiscMU on flat and hierarchical structure of compartments respectively.

Secreted

Root

Nuclear

分泌途径

Cytoplasm

线粒体

ER

细胞液

Golgi

过氧物酶体

Vacuole

细胞膜

Membrane

液泡

Peroxisome

Cytosol

Mitochondria

高尔基体

Secreted pathway

Intra cellular

Figure S1. Hierarchical (tree) structure of compartments based on cellular sorting.

- 1. Classification Accuracy


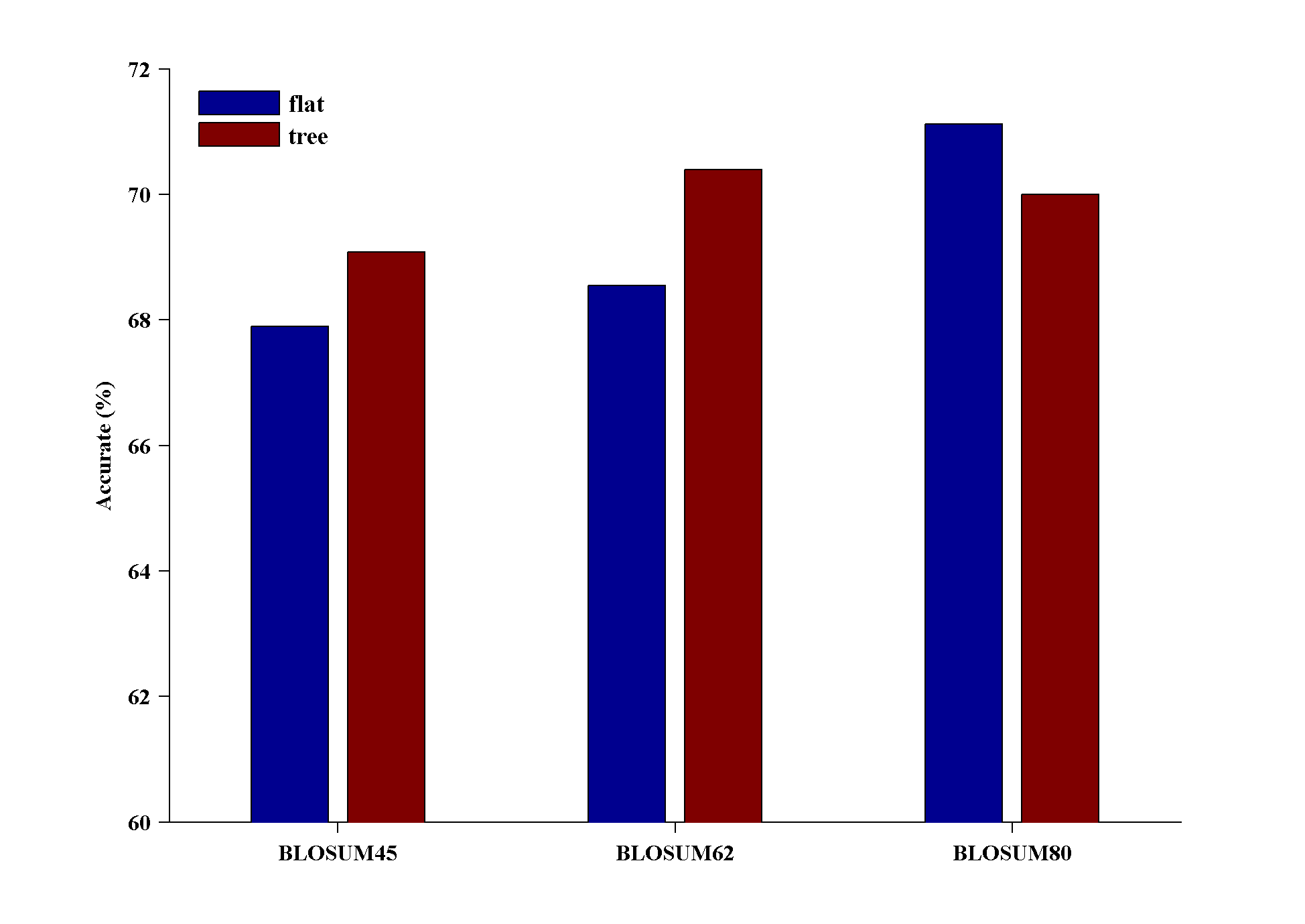


Figure S2. Accuracy of predictions based on simulated evolution by using BLOSUM45, BLOSUM62 and BLOSUM80 respectively.

The classification accuracy is obtained as follows: firstly, HMMs are trained on the PSLT2 dataset; then the sequences of PSLT2 are converted to feature vectors according to log-likelihood ratio; finally, the SVM is performed to classify the sequences. Here we don’t use the cross-validation or independent test set, so the classification accuracy is higher than the one presented in the paper. But it can also explain the problem to some extent. From Figure S2 we can see that the accuracy under BLOSUM80 with ﬂat structure is the highest and the one under BLOSUM62 with tree structure is lower than it, however, they differ by less than a percentage point. These may be due to that the BLOSUM80 makes the amino acid more conservative after substitution in the simulated mutation than BLOSUM62 and BLOSUM45, and this makes the risk of over-fitting increase. The higher prediction accuracy under BLOSUM80 may be caused by the over-fitting.

- 1. Known Motifs Recovery


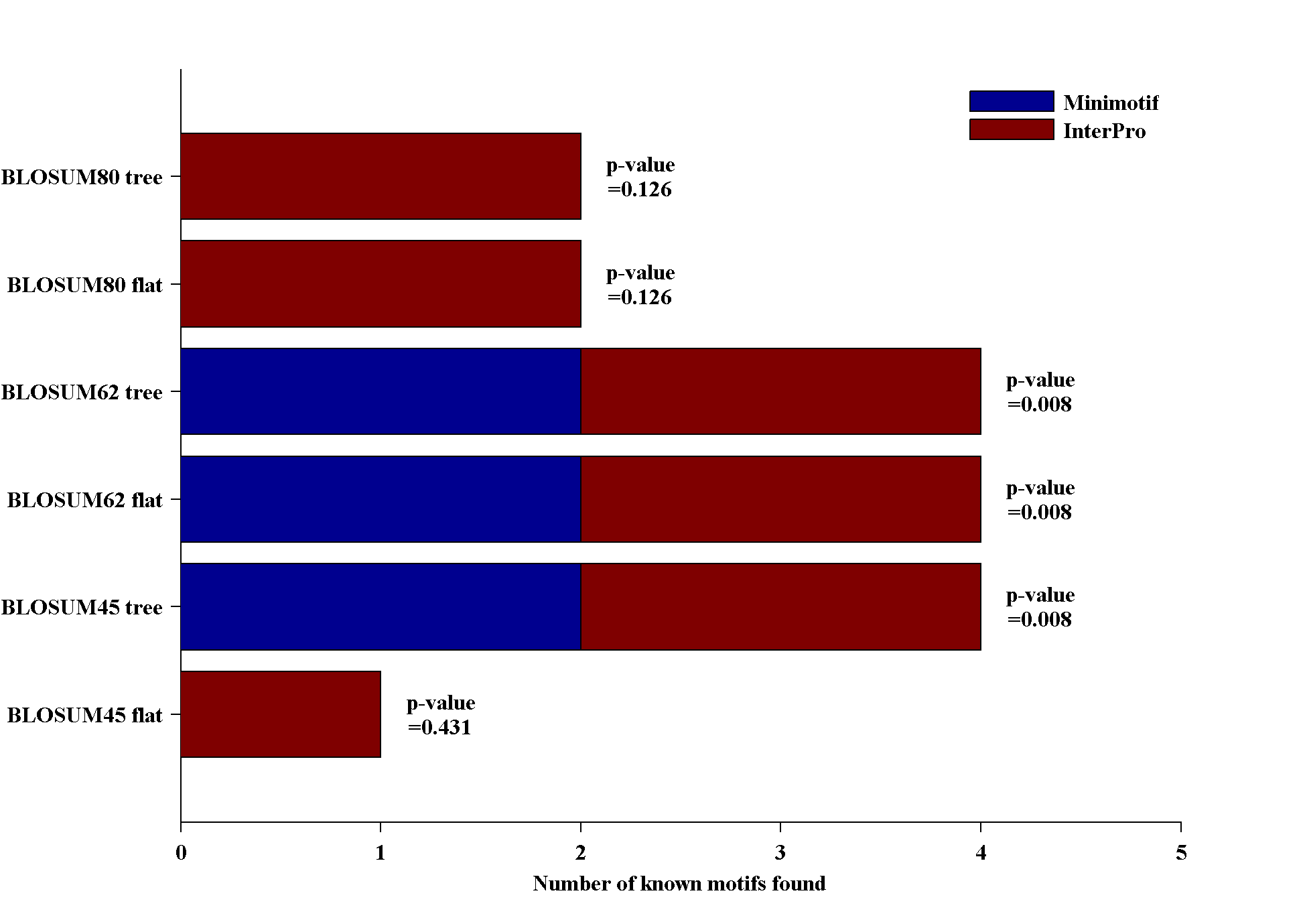


Figure S3. Number of known motifs recovered based on simulated evolution by using BLOSUM45, BLOSUM62 and BLOSUM80 respectively.

Figure S3 indicates that DiscMU by using BLOSUM62 is able to recover the most known motifs followed by BLOSUM45 with the tree structure. The reason to explain this is that simulated evolution by using BLOSUM62 may ensure both the conservation and diversity of the generated sequences, the conservation can ensure the motif discovery method to make found motifs more conversed and the diversity can make it identify multiple non-redundant motifs. Consequently, it is helpful to recover the known motifs.

- 1. Conservative Analysis


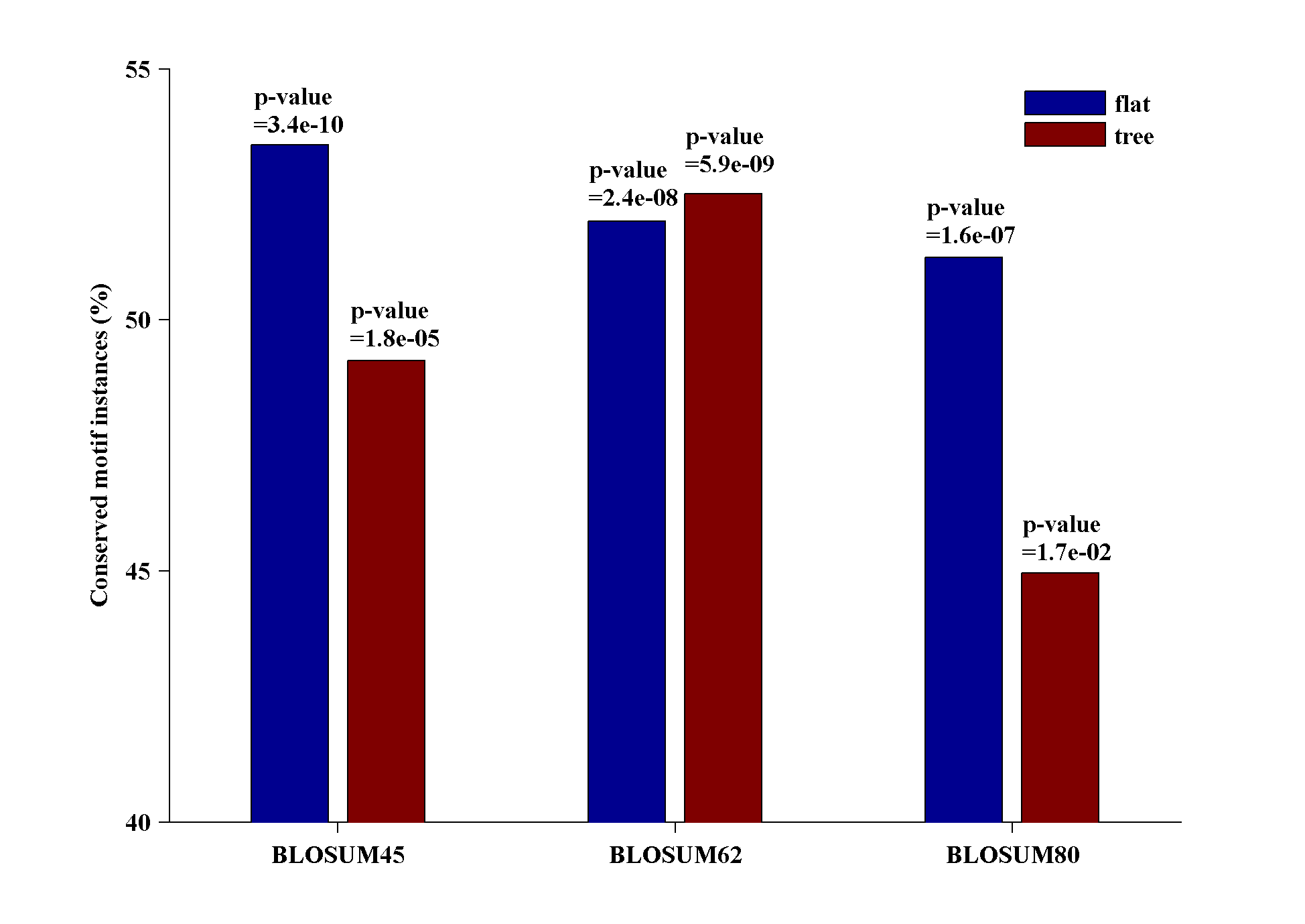


Figure S4. Percentage of conserved instances of the top 20 candidate motifs based on simulated evolution by using BLOSUM45, BLOSUM62 and BLOSUM80 respectively.

Figure S4 shows that DiscMU with flat structure by using BLOSUM45 reaches the highest percentage of conserved instances followed by BLOSUM62 with tree structure. However, they also differ by less than a percentage point. This may be caused by the diversity of the generated sequences brought by BLOSUM45 and BLOSUM62 and this makes the methods be beneficial to discovery multiple non-redundant motifs, and these found motifs may be potential sorting signals or involved in other functions that proteins in a given compartment need to carry out.

To sum up, the DiscMU method by using BLOSUM62 makes the percentage of conserved motif instances relatively high and statistically significance while ensuring relatively high prediction accuracy. At the same time, it recovers the most known motifs. However, the DiscMU method by using BLOSUM45 or BLOSUM80 can only guarantee one indicator of the classification accuracy and the percentage of conserved instances relatively good but not much.

1. Comparison of Known Motifs with Found Motifs

We compare the motifs found by DiscMU with the known motifs recovered by them. Known motifs collected from Minimotif Miner are experimentally validated to be involved in protein targeting, while the ones from InterPro are associated with localization. The evaluation criteria of whether an InterPro motif is considered to be associated with localization is that it occurs more than four times in one compartment and occurs in at most three compartments [1]. This evaluation process is executed by using the software InterProScan [2] to perform a filter step. Consequently, an InterPro motif may be associated with two or three distinct localizations (compartments).

Table S1 shows the recovered motifs of InterPro and the HMM logos of the found motifs, while Table S3 shows the known motifs of Minimotif Miner. As we mentioned in the paper, if the known motif instance is overlapped by the found motif instance at least half, it is considered to be correctly identified. And if one-third of the known motif instances are correctly identified when the number of predictions is four times the number of instances, this known motif is judged to be recovered.

Table S1. The known motifs of InterPro recovered by DiscMU.

| Compartment | Known | TP | Pred | True | Percent | Recall | Found HMM Logo |
| --- | --- | --- | --- | --- | --- | --- | --- |
| Cytosol | IPR000426 | 3 | 28 | 7 | 10.71 | 42.86 | 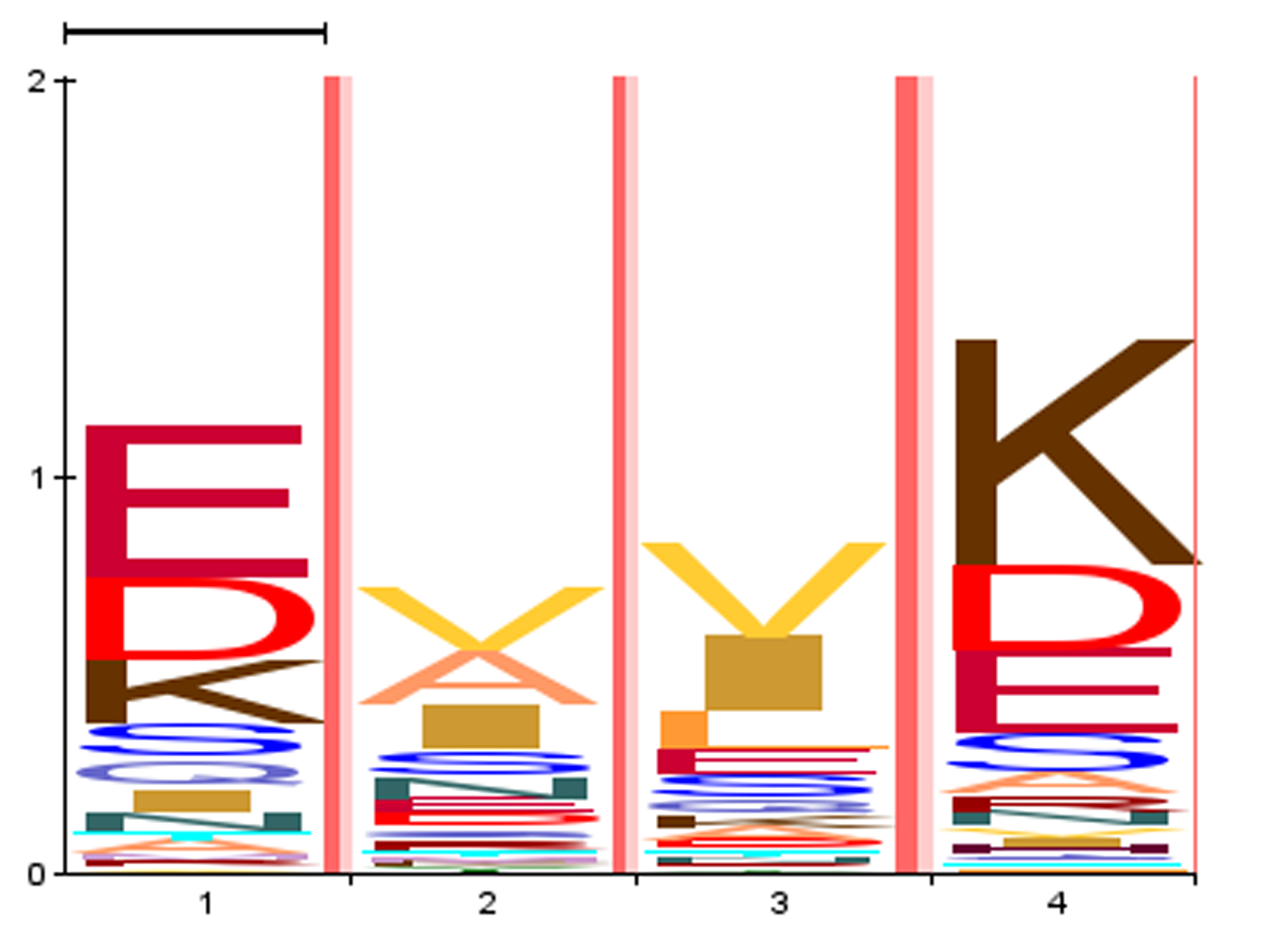 |
| ER | IPR000426 | 2 | 24 | 6 | 8.33 | 33.33 | 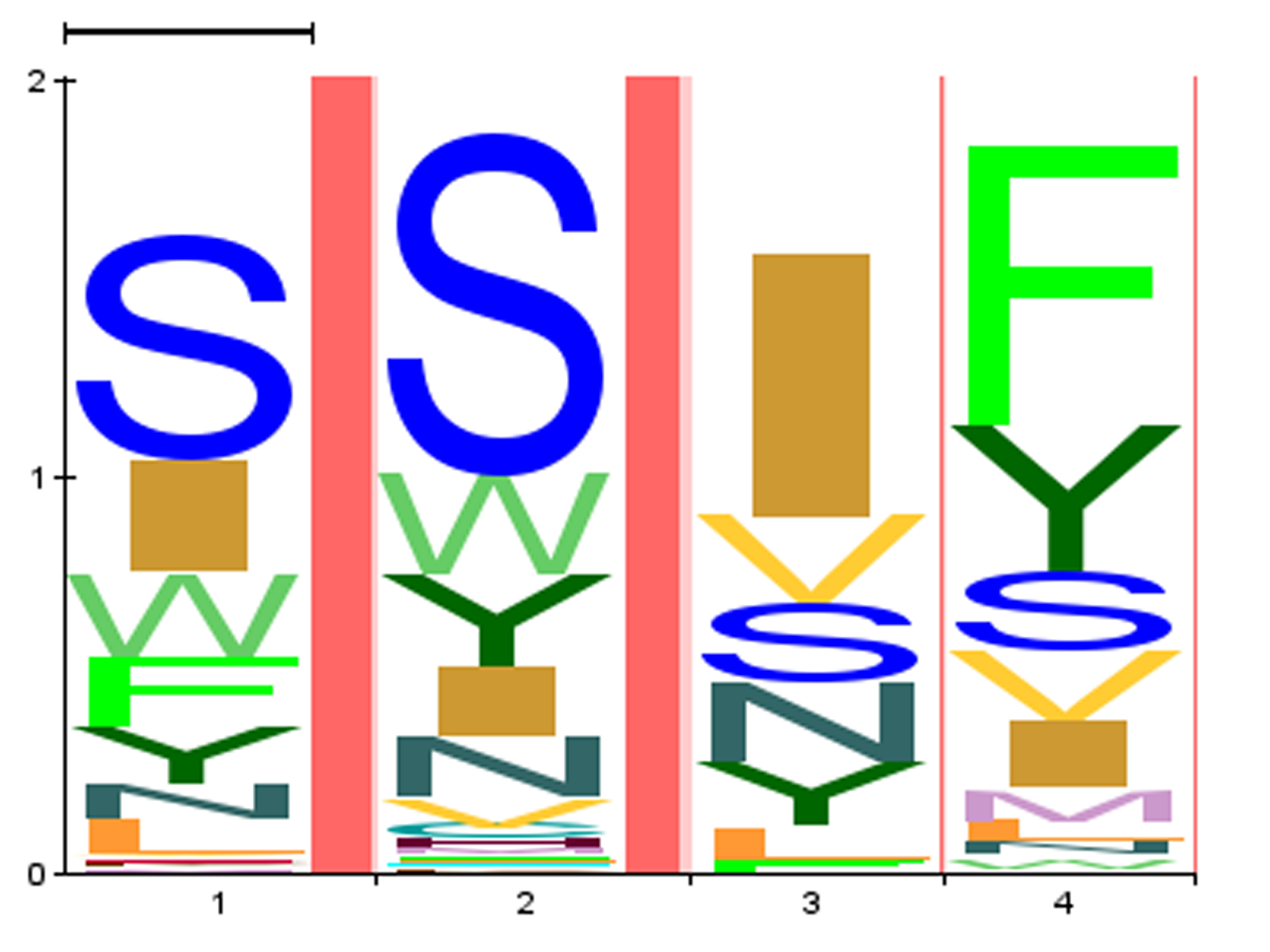 |
|  |  | 5 | 52 | 13 | 9.62 | 38.46 |  |

The first column is the cellular compartment associated with both the known motif and the found motif at the same time; the second column is the InterPro ID or regular expression of the known motif; the third column is the number of correctly identified instances of the known motif; the fourth column is the number of the found motif instances; the fifth is the number of the known motif instances; Percent = TP / Pred; Recall = TP / True; the last column is the HMM logo of the found motif. The last row shows the sum of TP, Pred and True respectively, and the Percent and Recall calculated by these sums. The regular expression of IPR000426 is [FYNAGS]-x(4)-[STNLV]-x-[FYW]-S-[PDS]-x(0,1)-G-[RKHDS]-x(2)-Q-[LIVA]- [DENR]-[YH]-[GSAD]-x(2)-[GSAG].

Table S2. The sequences that contain IPR00426.

| #Motif | Seq ID | Start | End | Compartments |
| --- | --- | --- | --- | --- |
| IPR000426 | YMR314W | 6 | 29 | Cytosol, Nuclear, ER |
| IPR000426 | YOL038W | 4 | 27 | Cytosol, Nuclear, ER |
| IPR000426 | YGR135W | 6 | 29 | Cytosol, Nuclear, ER |
| IPR000426 | YML092C | 5 | 28 | Cytosol, Nuclear, ER |
| IPR000426 | YOR362C | 8 | 31 | Cytosol, Nuclear, ER |
| IPR000426 | YGR253C | 8 | 31 | Cytosol, Nuclear, ER |
| IPR000426 | YGL011C | 12 | 35 | Cytosol, Nuclear |

The columns “Start” and “End” are the start and end positions of IPR00426 in sequences; the last column is the cellular compartments that the sequence located in, as we described in section “Datasets”, several proteins are localized to more than one compartment.

According to the InterPro motif collecting process, IPR00426 was considered associated with Cytosol, Nuclear and ER. The sequences that contain IPR00426 are shown in Table S2. The HMM logos of Table S1 reflect that the front half part of IPR000426 partially matches the HMM found for endoplasmic reticulum (ER) and the second half part of it partially matches the HMM found for cytosol. Note that not all found motifs are necessarily relevant to localization. Many of them may be involved in other functions that proteins in a given compartment need to carry out or reflect differences in amino acid composition between protein sequences with different subcellular localizations.

Table S3. The known motifs of Minimotif Miner recovered by DiscMU.

| Compartment | Known | TP | Pred | True | Percent | Recall | Found HMM Logo |
| --- | --- | --- | --- | --- | --- | --- | --- |
| Golgi | WW | 3 | 28 | 7 | 10.71 | 42.86 | 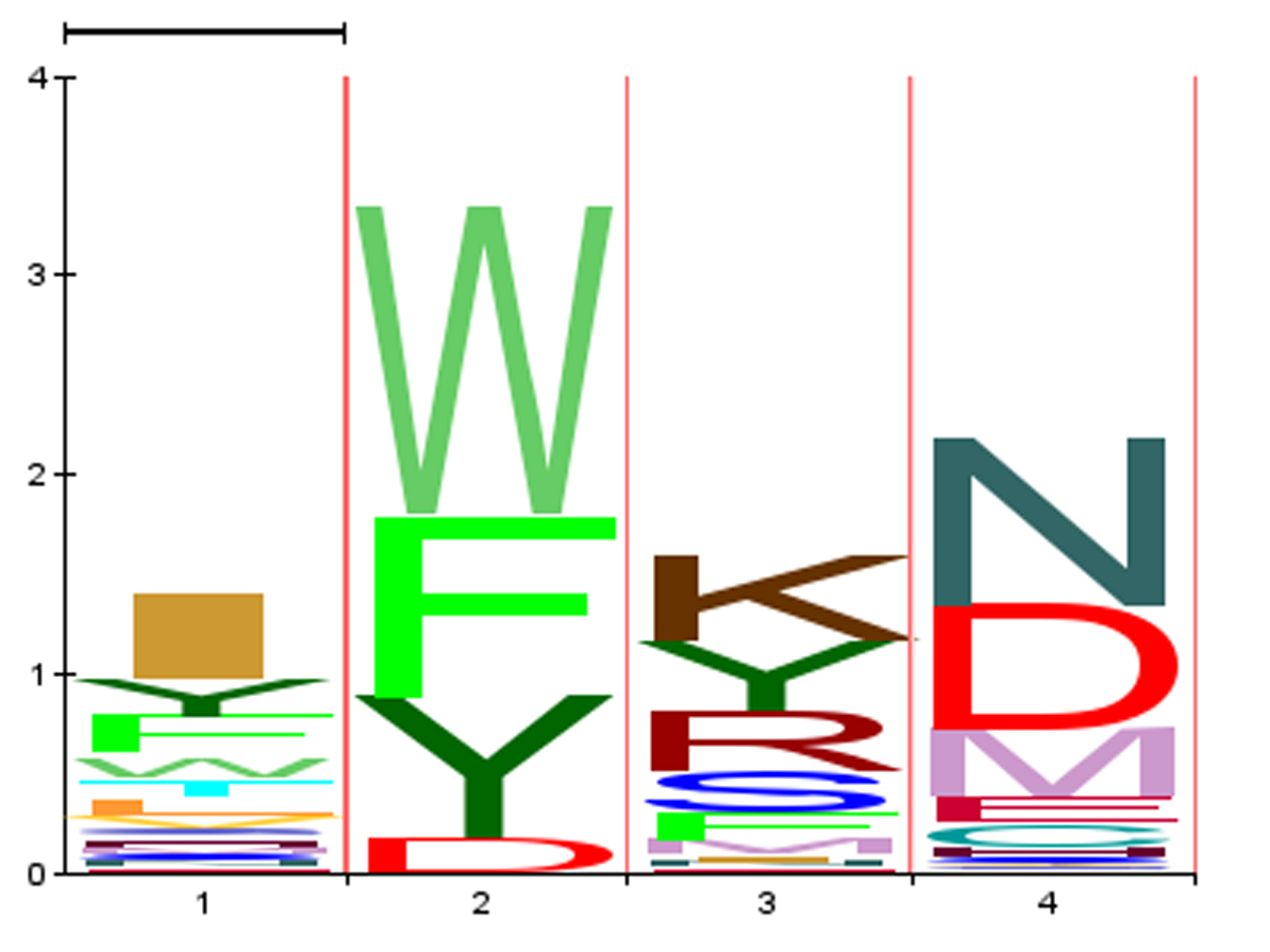 |
| Nuclear | [KR]{4} | 104 | 764 | 191 | 13.61 | 54.45 | 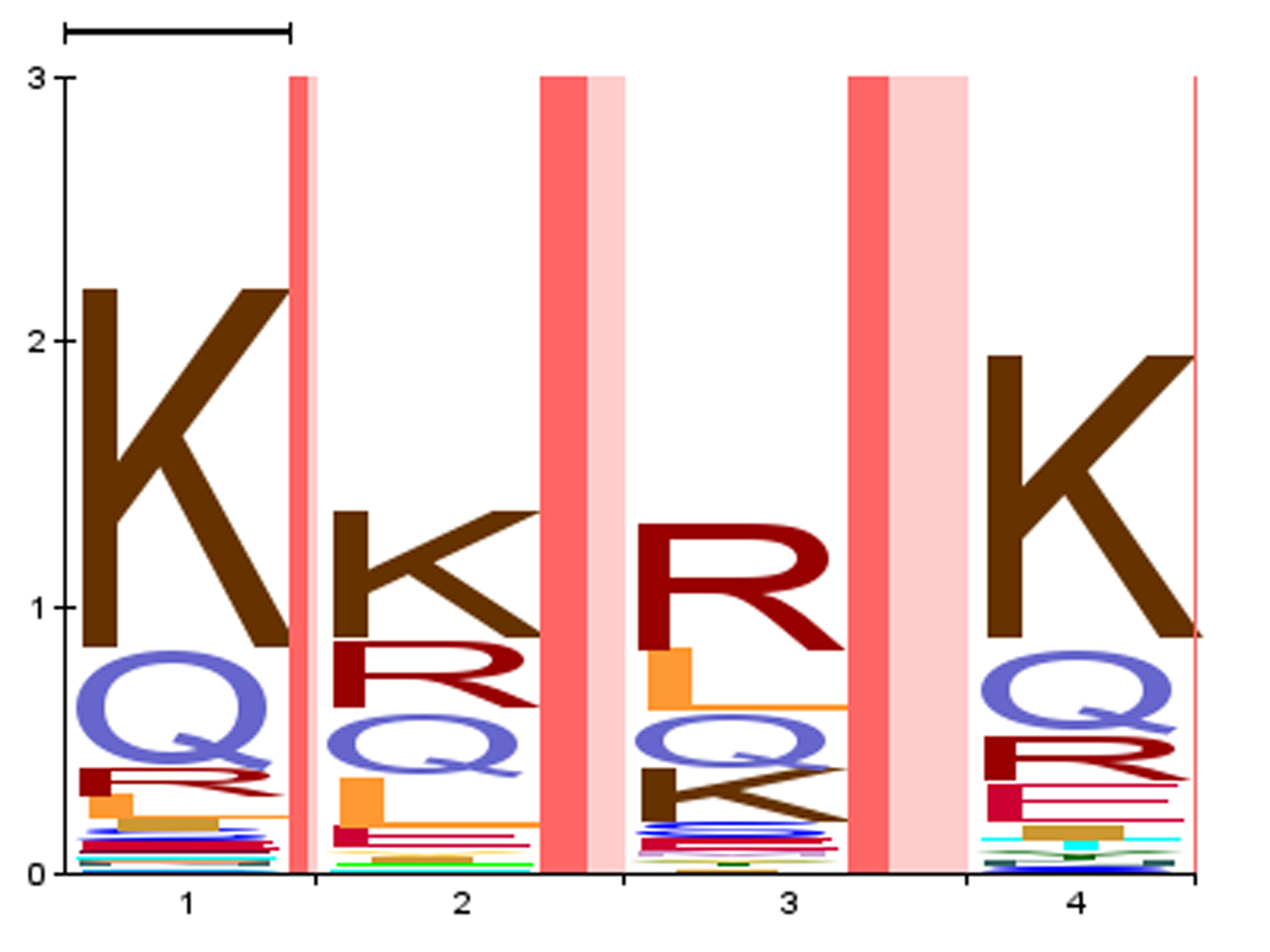 |
|  |  | 107 | 792 | 198 | 13.51 | 54.04 |  |

Table S3 shows that the WW partially matches the HMM found for Golgi and the [KR]{4} partially matches the HMM found for nuclear. Here, the short pattern WW seems more likely to be overlapped, since we set the threshold as “at least half” as described above and a single Tryptophan will be enough to pass it for WW. However, for many longer and more complex patterns it is hard to exceed this threshold. So that setting a stricter threshold such as “at least three-quarter” or “totally” may be of little significance. Meanwhile, some less strict thresholds such as “at least one-quarter” [3] and “at least 1 nt” [4] have been used for DNA sequences and it has been shown that these thresholds were already relatively appropriate in analysis of comparing the found motifs with the known motifs. Consequently, the threshold “at least half” used in our paper may be relatively appropriate for protein sequences.

In summary, when the length of the known motifs is around 4 (the number that we set for the match states of HMM), the regular expressions of the known motifs recovered match the found HMMs relatively better, otherwise they are not good and especially when the length of the known motifs is particularly long. Therefore, finding the optimal motif length is very important in motif discovery. We will consider this problem in future research.

1. Statistical Significance

Here, we describe the statistical significances (p-value) of the known motifs recovered and the discovered motif conservation which were used by Lin et al [1].

The statistical significance (p-value) of recovering *x* known motifs is calculated as the number of random motif sets (each set contains 90 randomly generated profile HMMs) that recovered *x* or more known motifs divided by 1,000 (the total number of random motif sets). For example, the method DiscMU recovered four known motifs, and eight random motif sets out of 1,000 recovered four or more known motifs, so the p-value is estimated as 0.008. The randomly generated profile HMM is obtained by the following process: First, a random 4-mer is generated using a uniform distribution among the 20 amino acids. Then, an HMM is constructed and the emission probabilities of the match states is estimated with the assumption that this 4-mer is observed 10 times with a pseudocount of 1. Other emission and transition probabilities are set to default values of HMMER.

The statistical significance (p-value) of motif conservation is calculated as follows: The number of conserved 4-mer and total possible 4-mers is obtained by scanning through all protein sequences using a sliding window of length four, where conserved 4-mers are identified by ClustalW as described in the main text. For each motif discovery method, the number of conserved motif instances and the total number of top motif instances is collected, and then the percentage of conserved motif instances is calculated. The p-values are calculated by hypergeometric test with the null hypothesis that the percentages of conserved motif instances and conserved 4-mers are same and the alternative hypothesis that the percentage of conserved motif instances is higher than that of conserved 4-mers.

References

1. Lin, T. H., Murphy, R. F., & Bar-Joseph, Z. (2011). Discriminative motif finding for predicting protein subcellular localization. Computational Biology and Bioinformatics, IEEE/ACM Transactions on, 8(2), 441-451.
2. Zdobnov, E. M., & Apweiler, R. (2001). InterProScan–an integration platform for the signature-recognition methods in InterPro. Bioinformatics, 17(9), 847-848.
3. Tompa, M., Li, N., Bailey, T. L., Church, G. M., De Moor, B., Eskin, E., ... & Zhu, Z. (2005). Assessing computational tools for the discovery of transcription factor binding sites. Nature biotechnology, 23(1), 137-144.
4. Hu, J., Li, B., & Kihara, D. (2005). Limitations and potentials of current motif discovery algorithms. Nucleic acids research, 33(15), 4899-4913.
